# Intermittent white light at 60Hz, a novel non invasive brain stimulation (NIBS), modulates neuroplasticity and ameliorates depressive-like symptoms in animal models - preliminary preclinical and in human data

**DOI:** 10.1192/j.eurpsy.2025.794

**Published:** 2025-08-26

**Authors:** M. T. Ferretti, A. Venturino, M. Alamalhoda, S. Gorkiewicz, M. Muhia, S. Siegert

**Affiliations:** 1 Syntropic Medical; 2 ISTA, Klosterneuburg, Austria

## Abstract

**Introduction:**

Non-invasive brain stimulation (NIBS) is emerging as a promising option for the treatment of psychiatric diseases, including major depressive disorder (MDD). In this context intermittent white light at a specified frequency holds high promise. We have previously shown that 60Hz stimulation in mice induces selective brain entrainment associated with microglia-mediated remodeling of the perineuronal nets (Venturino et al., Cell Reports 2021).

**Objectives:**

Here, we extend our previous findings with 60Hz stimulation to assess behavioral effects in mice and EEG response in healthy volunteers.

**Methods:**

For the preclinical data, we exposed C57Bl6/J mice to a battery of behavioral tests to assess anxiety level, learning capability, and response to various stress paradigms after 60Hz light (2h per day/5 days) compared to constant light. Weight change, water and food intake were recorded. For human studies, a cohort of 12 healthy volunteers (6M, 6F) was recruited; their EEG response was investigated with an 8-channel EEG setup following acute (same day), short (5 days), and intermediate (3 weeks) stimulation with 60Hz entrainment (n=6) or sham light (n=6).

**Results:**

Preliminary data from the preclinical behavior studies indicate that 60Hz treatment improves the social interaction of socially defeated mice compared to sham light stimulation. Furthermore, the animals showed less anxiety-related behavior when exposed to the elevated plus maze. No differences were noticed in weight change, water and food intake following 60Hz stimulation.

In healthy volunteers, we observed robust and widespread entrainment at 60Hz after acute 60Hz stimulation; the entrainment spread beyond the visual cortex and reached the frontal cortex. The normalized power of the 60Hz component slightly declined over time but remained significant as compared to sham stimulation at three weeks, indicating sustained EEG response. The stimulation was very well tolerated overall, without major side effects.

**Conclusions:**

60Hz intermittent light induces strong and sustained neuronal response in mice and humans, is well tolerated, and ameliorates depressive-like symptoms in the social defeat model in mice. 60Hz might represent a novel NIBS for the treatment of psychiatric disorders, including MMD.

**Disclosure of Interest:**

None Declared

